# Fast Wound Healing with a New Functional Hyaluronic Acid Dual Network Hydrogel

**DOI:** 10.3390/gels11040266

**Published:** 2025-04-03

**Authors:** Lichun Wu, Yu Zhou, Yi Zhang, Jia Hu, Yasuhiro Ikegami, Shinichi Aishima, Hiroyuki Ijima

**Affiliations:** 1 Department of Chemical Engineering, Faculty of Engineering, Graduate School of Engineering, Kyushu University, 744 Motooka, Nishi-ku, Fukuoka 819-0395, Japan; wu.lichun.820@s.kyushu-u.ac.jp (L.W.); zhou.yu.222@s.kyushu-u.ac.jp (Y.Z.); hu.jia.511@s.kyushu-u.ac.jp (J.H.); yikegami@chem-eng.kyushu-u.ac.jp (Y.I.); 2 Institute for Materials Chemistry and Engineering, Kyushu University, 744 Motooka, Nishi-ku, Fukuoka 819-0395, Japan; zhang_yi@ms.ifoc.kyushu-u.ac.jp; 3 Department of Scientific Pathology, Graduate School of Medical Sciences, Kyushu University, 3-1-1 Maidashi, Higashi-ku, Fukuoka 812-8582, Japan; aishima.shinichi.476@m.kyushu-u.ac.jp

**Keywords:** hyaluronic acid, double network hydrogels, wound healing

## Abstract

As dressings for moist wound healing, hyaluronic acid hydrogels play a significant role in maintaining moisture and promoting wound healing. However, existing hydrogel dressings are inadequate in terms of slow gelation time, weak mechanical performance, and fast degradation, which increases the risk of secondary infections during treatment. Therefore, we developed a hyaluronic acid double network hydrogel (DNH). Compared to single-network hydrogels (hydrazone and Diels–Alder), DNH shows a short gelation time (25 s) and strong mechanical properties (Young’s modulus = 82 kPa). These advantages enable DNH to immediately fill the irregular shape of the wound after gelation and remain intact after being squeezed. Swelling tests indicated that DNH had a suitable swelling ratio and maintained its structural integrity after swelling. We evaluated the use of DNH as a moist dressing for full-thickness wound healing in vivo. DNH-treated wounds healed faster, with enhanced blood vessel formation and macrophage polarization than gauze-treated wounds. These findings suggest that DNH not only accelerates wound healing but also improves tissue regeneration. Therefore, DNH may be a suitable moist dressing for wound healing.

## 1. Introduction

Skin is the first line of defense in animals and protects the body from environmental damage. However, the skin is vulnerable to being damaged by injury, surgery, and disease [1]. Appropriate wound dressings are essential for both surgical wounds and daily life. Conventional gauze bandages are used for wound treatment because of their ability to absorb exudates. However, it often causes immense pain owing to the adhesion between the wound scabs and the gauze network, causing inflammation, which delays wound healing [1]. With growing concerns about wound care, it is essential to develop effective and comfortable dressings for wound healing. Hydrogel wound dressings are promising materials in wound care to keep wound moisture, absorb excess exudate, transfer oxygen, provide protection, and promote skin regeneration [2,3,4].

Natural polysaccharides (hyaluronic acid (HA) [5,6], chitosan [4,7], and alginate [8]) and proteins (e.g., gelatin [2,7], silk fibroin [6], and albumin [9]) have been widely reported as wound dressing materials owing to their biocompatibility, biodegradability, and physical tunability. HA was used to prepare the hydrogels because of its unique chemical structure and biofunction in wound healing. HA is a linear glycosaminoglycan with a large number of modified groups, such as carboxyl and hydroxyl groups, which facilitate the development of functional hydrogels by HA chemical modification. During wound healing, HA, an important component of the extracellular matrix (ECM), can modulate inflammation [10], promote angiogenesis, and accelerate collagen deposition [5,11]. In addition, HA hydrogels show excellent properties in absorbing excess wound exudate and keeping the wound moist because of their high hydrophilicity [12]. However, most HA hydrogels exhibit fast degradation and poor mechanical properties [13]. To solve this problem, researchers have developed functional HA hydrogels with the required mechanical properties and good healing efficiency [14,15].

The double network hydrogel (DNH) is an inter-crosslinking network consisting of two interpenetrated networks [16]. Compared to single-network HA hydrogels, two-covalent networks have proven to be promising materials with tunable mechanical properties [17,18]. HA derivatives provide additional options for the preparation of hydrogel [5,11]. Recently, various DNH based on HA derivatives have been used to develop hydrogels with strong mechanical properties, such as a combination of hydrazone crosslinking and Diels–Alder crosslinking (methyl-gifted HA) [17], hydrazone crosslinking (aldehyde-added HA), and photo-crosslinking [18]. Efficient exudate absorption and structural stability are critical for effective wound healing.

Based on the above considerations, we fabricated DNH composed of HA derivatives. In this double-crosslinking system, the hydrazone hydrogel (HYH) was crosslinked via hydrazone bonds using oxidized HA and adipic dihydrazide (ADH), which provided fast gelation to DNH [11,19,20]. HA with a furan group was crosslinked with maleimide groups to form a Diels–Alder hydrogel (DAH) to enhance its mechanical properties. To verify this, the structural, mechanical, and swelling properties of three types of hydrogels (HYH, DAH, and DNH) were investigated. Finally, DNH was applied to a wound to assess its ability to promote full-thickness wound healing based on the wound closure rate and histological observations. We expected DNH to maintain wound moisture, absorb exudates, and accelerate wound healing.

## 2. Results and Discussion

### 2.1. Synthesis and Characterization of DNH

To design a DNH with suitable gelation time and sufficient mechanical properties, different ratios of raw materials were investigated, as shown in Table S1. Based on the gelation time of the single-network hydrogel, ADH was added sooner or later after the other components to uniformly form DNH.

**Figure 1:**
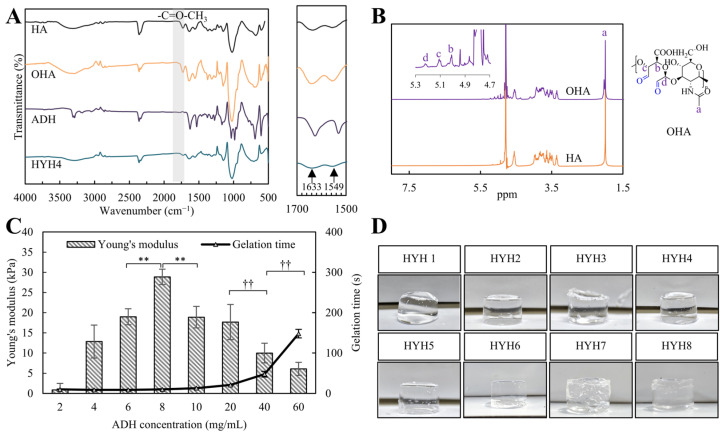
Properties of HYH. (**A**) ATR-FTIR spectra of HA, OHA, ADH, and HYH. (**B**) ^1^H-NMR of HA and OHA. (**C**) Young’s modulus and gelation time of HYH (*n* = 3, the value indicates mean ± S.D.). (**D**) Appearance of HYH with increasing concentration of ADH. Significance was assessed using one-way ANOVA with Tukey’s multiple comparisons test, giving *p* values, ** *p* < 0.01 of Young’s modulus, ^††^ *p* < 0.01 of the gelation time.

The formation of a hydrazone bond was confirmed by an FTIR spectrum (peaks at −1549 cm^−1^ and 1633 cm^−1^), and the newly formed aldehyde was confirmed by ^1^H-NMR (peaks at 5.01 ppm, 5.11 ppm, and 5.21 ppm from OAH, as in Figure 1A,B, confirming the successful formation of HYH. The optimized formulation, HYH4 (with a volume ratio of OHA to ADH of 4:1), was obtained using an ADH concentration of 8 mg/mL owing to the shortest gelation time (10.00 ± 1.73 s) and the highest Young’s modulus (28.87 ± 1.92 kPa), as shown in Table S2 and Figure 1C. HYH4 exhibited a transparent appearance, which is beneficial for wound observation during the healing process. Finally, HYH4 was used to prepare the DNH.

**Figure 2:**
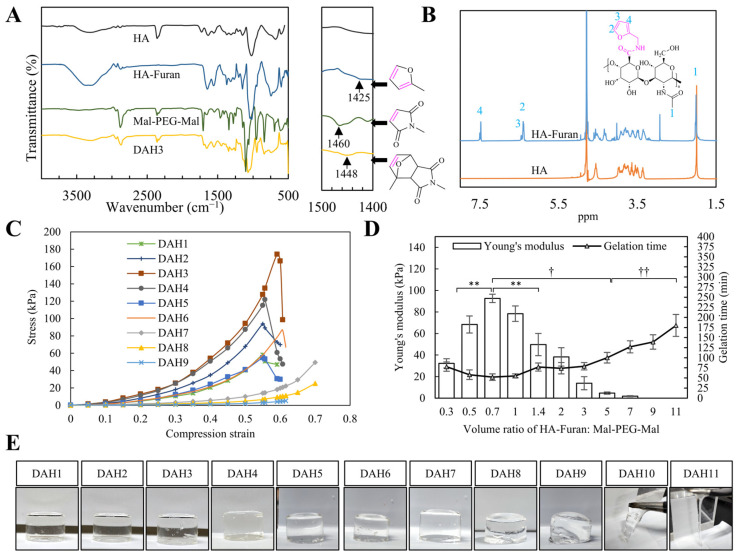
Properties of DAH. (**A**) ATR-FTIR spectra of HA, HA-Furan, Mal-PEG-Mal, and DAH3. (**B**) ^1^H-NMR of HA and HA-Furan. (**C**) Compressive stress–strain curves for DAH. (**D**) Young’s modulus and gelation time of DAH (*n* = 3, the value indicates mean ± S.D.). (**E**) Appearance of DAH with different volume ratios between HA-Furan and Mal-PEG-Mal. Significance was assessed using one-way ANOVA with Tukey’s multiple comparisons test, giving *p* values, ** *p* < 0.01 of Young’s modulus, ^†^ *p* < 0.05 of the gelation time, ^††^ *p* < 0.01 of the gelation time.

The formation of DAH was confirmed by FTIR and ^1^H-NMR spectroscopies, as shown in Figure 2A,B. In the FTIR spectrum, the disappearance of the C=C signals indicated the consumption of furan and maleimide groups. A new peak emerges at 1448 cm^−1^, which corresponds to the C=C bond in the Diels–Alder adduct. The peaks at 6.43, 6.56, and 7.57 ppm in the ^1^H-NMR spectrum correspond to the protons of the furan groups, confirming the successful grafting of furan onto HA. The substitution degree of HA-Furan was determined to be 69.5% ± 1.5%. The volume ratios of HA-Furan and Mal-PEG-Mal significantly affected compression properties. When DAH3 was obtained with a volume ratio of HA-Furan to Mal-PEG-Mal of 0.7, as shown in Figure 2C,D, the maximum yield strength was 174.23 kPa under 59% compression strain, and Young’s modulus also reached the highest value (92.62 kPa). Moreover, the gelation time of DAH also decreased to 52 ± 8 min under the volume ratio of HA-Furan to Mal-PEG-Mal of 0.7 from 78 ± 12 min under the volume ratio of HA-Furan to Mal-PEG-Mal of 0.3. Young’s modulus decreased, whereas the gelation time increased significantly as the volume ratio increased from 0.7 to 11. Compared with maleimide groups, excess furan groups are safe for biological application [16]. Therefore, DAH3, DAH4, DAH5, DAH6, and DAH7 were selected for further investigation of DNH. All DAH samples were transparent, which is advantageous for observing the wound healing process, as shown in Figure 2E.

**Figure 3:**
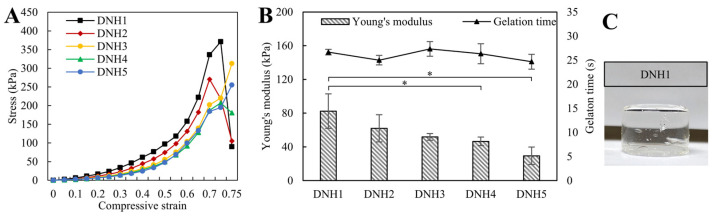
Properties of DNH. (**A**) Compressive stress–strain curves. (**B**) Young’s modulus and gelation time (*n* = 3, the value indicates mean ± S.D.). (**C**) Appearance of DNH1. Significance was assessed using one-way ANOVA with Tukey’s multiple comparisons test, giving *p* values, * *p* < 0.05 of the Young’s modulus.

Following the optimization of HYH and DAH gelation, the results for DNH prepared with different HA-Furan and Mal-PEG-Mal ratios are shown in Figure 3. The results showed that the mechanical properties of DNH could be adjusted by changing the volume ratio of HA-Furan and Mal-PEG-Mal. DNH1 had the highest ultimate yield strength (371.25 kPa) under 71% compressive strain (Figure 3A). This is higher than that when acrylamide/N,N′-methylene bisacrylamide (242.4 ± 2.43 kPa) is used as wound healing hydrogel [21]. This indicates that DNH1 possesses sufficient mechanical properties for wound healing. The Young’s modulus of DNH1 (82.33 ± 26.67 kPa, Figure 3B) was between the HYH4 (28.87 ± 1.92) and DAH3 (92.62 ± 3.88 kPa). The DNH gelation time (25 s) did not change significantly with DAH variation. Although the time was longer than that of HYH4 (10.00 ± 1.73 s), it was still acceptable for the in situ gelation for wound healing and less than that reported by Fang (30 s) [22]. DNH was transparent, as shown in Figure 3C, which facilitated wound observation during healing. Based on its gelation properties, DNH1 was selected for further investigation of wound healing.

### 2.2. Swelling Rate, Resilience, and Morphology of Hydrogel

**Figure 4:**
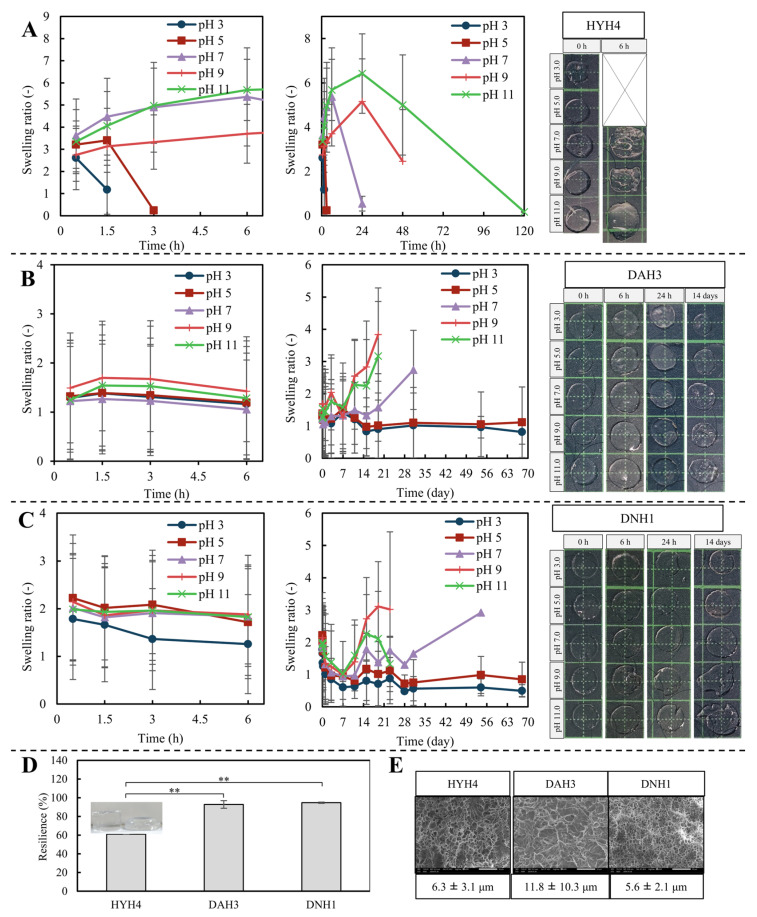
Swelling rate and related appearance of the swollen hydrogel at different pH values (*n* = 3, the value indicates mean ± S.D.). (**A**) HYH4. (**B**) DAH3. (**C**) DNH1. (**D**) Resilience of the hydrogel (*n* = 3, the value indicates mean ± S.D.). (**E**) SEM images of cross-section hydrogel and the corresponding mean diameter (*n* = 10, the value indicates mean ± SD.). Significance was assessed using one-way ANOVA with Tukey’s multiple comparisons test, giving *p* values, ** *p* < 0.01.

Healthy skin has a slightly acidic pH, whereas wounds are mainly alkaline [23,24]. Thus, hydrogel swelling properties were investigated at different pH values. As shown in Figure 4, HYH4, DAH3, and DNH1 had different swelling properties. HYH4 absorbed phosphate-buffered saline (PBS) at a maximum of 5.42 times its weight at pH 11. However, it was completely degraded after 6 h at pH 3.0 and 5.0. The swelling rate of HYH under acidic conditions was higher than that under alkaline conditions, which resulted from the hydrolysis of hydrazone [25]. In addition, the degradation rate was higher than the swelling rate, leading to faster degradation [26]. DAH3 had a low swelling ratio under acidic conditions and fast swelling under alkaline conditions, which can be attributed to the opening of the furan rings under alkaline conditions [27]. When DNH1 was tested, the hydrogel maintained a swelling ratio close to 2 from pH 5.0 to 11.0 within the first 6 h, which demonstrated that DNH absorbed an equivalent volume solution over a wide pH range (pH 5.0–11.0). This range fully covers the skin pH range (pH 5.0–9.0). Therefore, DNH1 easily satisfies the requirements for absorbing exudates while maintaining its structural integrity. In summary, compared with HYH and DAH, DNH1 exhibited optimal swelling properties, ensuring efficient absorption of the wound exudate while maintaining its original shape after swelling.

The elasticity of the hydrogel can protect the wound from rupture owing to compression or movement of the body. To further investigate the elasticity of the hydrogels, they were compressed six times under 60% compression strain to test their ability to recover their original form. As shown in Figure 4D, HYH4 deformed severely but did not break, indicating that HYH4 had weak elasticity, which can be attributed to its dynamic hydrazone bonds. DAH3 and DNH1 exhibited excellent elasticity. This can be attributed to the flexible PEG linkages in HYH and DNH [28]. Pore size analysis revealed that HYH and DNH had smaller pore sizes than DAH (Figure 4D). The smaller pore size of DNH1 facilitated the absorption of wound exudates while maintaining the moisture content of the hydrogel, which is beneficial for wound healing.

### 2.3. Cytocompatibility of DNH1

**Figure 5:**
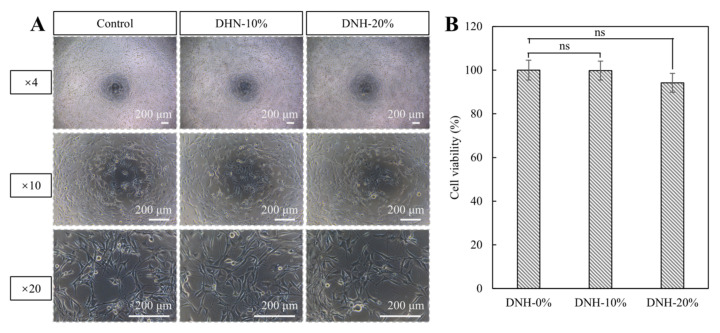
(**A**) The morphology of NIH/3T3 cells. (**B**) The cell viability ratio of NIH/3T3 cells. Significance was assessed using one-way ANOVA with Tukey’s multiple comparisons tests (*n* = 3, the value indicates mean ± S.D.); ns represents no significance.

The cellular-level biocompatibility of DNH1 was evaluated using NIH/3T3 fibroblast cells before wound healing in vivo. As shown in Figure 5A, cells treated with DNH1 exhibited normal adhesion and spreading, with a morphology similar to that of the control group, indicating no apparent cytotoxic effects. Cell viability assays confirmed that DNH-treated cells maintained over 95% viability, even at concentrations up to 20% (*v*/*v*). There were no significant differences in cell viability between the treated and control groups, suggesting that DNH1 does not adversely affect cell proliferation. These results demonstrate that DNH1 is biocompatible and suitable for wound healing applications, providing a safe microenvironment for fibroblast growth.

### 2.4. Wound Healing with DNH

**Figure 6:**
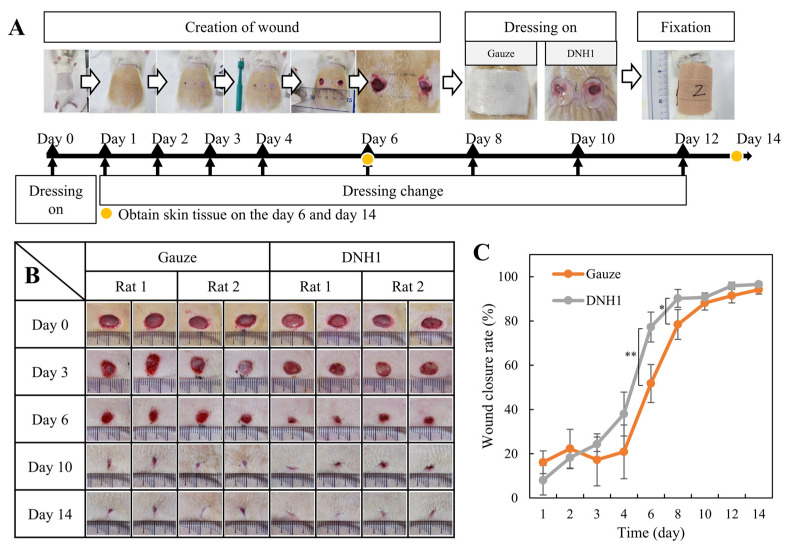
Wound healing procedure. (**A**) Detailed stage of wound healing. (**B**) Appearance of the wound treated with gauze and DNH1 on days 0, 3, 6, 10, and 14. (**C**) Quantification of wound closure (*n* = 3, the value indicates mean ± S.D.). Significance was assessed using one-way ANOVA with Tukey’s multiple comparisons test, giving *p* values, * *p* < 0.05, ** *p* < 0.01.

The healing capability of the hydrogel was also evaluated. Figure 6A displays the gross wound appearance on days 0, 3, 6, 10, and 14, indicating complete wound closure with DNH1 treatment for 2 weeks. A comparison using gauze is shown in Figure 6B. It can be seen that the wound treated with gauze remained visible on day 14. However, DNH1-treated wounds were nearly healed. This demonstrated faster wound healing with DNH. Further analysis (Figure 6C) shows no significant differences within the first 3 days. Between days 3 and 8, the wound closure rate with DNH was significantly higher than that with gauze treatment. Importantly, on day 8, the wound closure rate reached 91% with the DNH1 treatment, whereas the wound closure rate with gauze was only 78%. On day 10, wound closure rates in both groups were comparable (92%). These results indicated that DNH1 treatment shortened the wound healing time.

**Figure 7:**
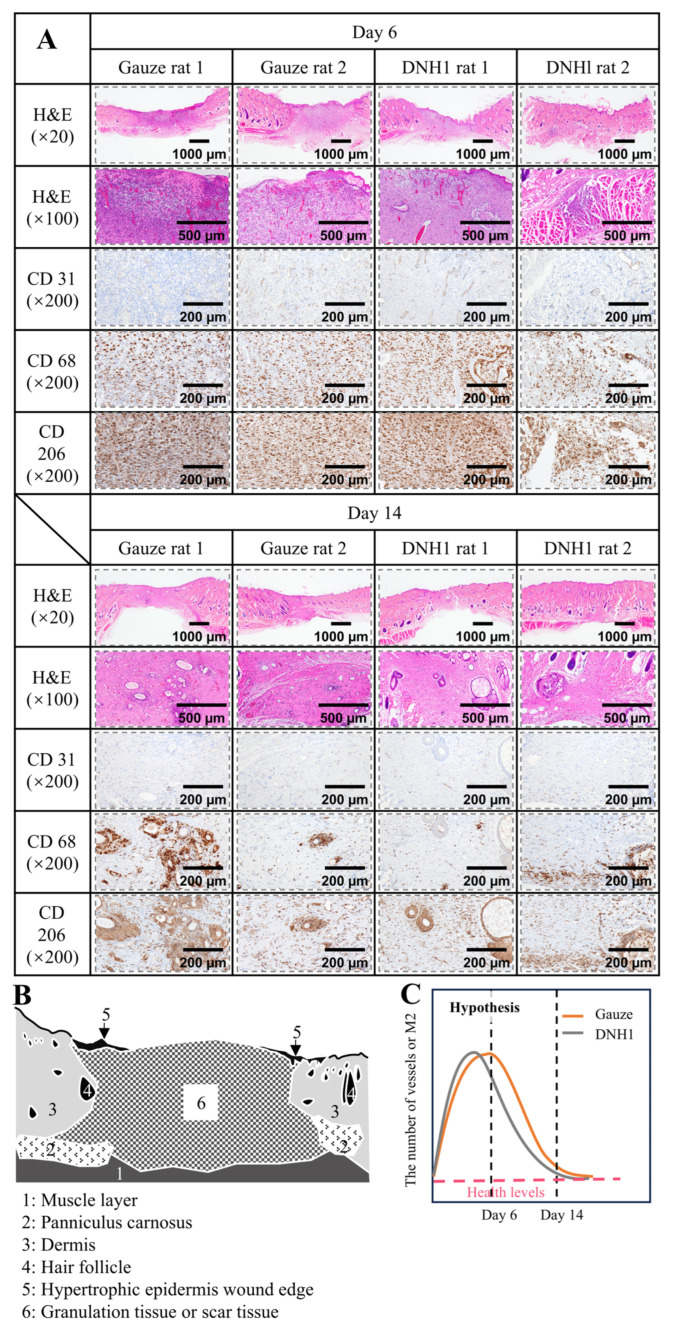
Histological evaluations of wounds. (**A**) Histological evaluations at 6 and 14 days. Scale bars, 1000 μm for H&E (×20), 500 μm for H&E (×100), and 200 μm for CD 31, CD68, and CD 206 (×200). (**B**) Wound structure. (**C**) Hypothesis of wound healing.

Tissue sections from the wound center were stained and analyzed on days 6 and 14, as shown in Figure 7. On day 6, the wounds treated with gauze exhibited a larger granulation area with significant immune cell infiltration and lacked the formation of skin appendages such as hair follicles and pores (Figure 7A,B). In contrast, the wounds treated with DNH1 displayed characteristics of the wound maturation stage, with a small number of hair follicles and pores. However, no significant differences in granulation epidermis and dermis thickness were observed between the gauze and DNH1 groups on day 6 (Table 1).

By day 14, the wounds in all groups had generally healed based on hematoxylin and eosin (H&E) staining (Figure 7A). The granulation epidermis thickness had reached a healthy skin epidermis range (41.97 ± 12.70 μm), indicating complete re-epithelialization. Importantly, the wounds treated with DNH1 exhibited smaller granulation areas and more mature hair follicles and pores than those treated with gauze. This H&E staining result was consistent with the wound closure rate results shown in Figure 6C, demonstrating that DNH1 not only accelerated wound closure but also improved the overall quality of healing by promoting skin appendage regeneration.

The number of vessels and macrophages on days 6 and 14 were recorded. M1 macrophages are angiogenesis initiators and secrete inflammatory cytokines [29], whereas M2 macrophages support angiogenesis to turn into mature vessels and secrete anti-inflammatory cytokines [29]. The number of CD68- (total macrophages) and CD206-positive (M2 macrophages) vessels were analyzed under the same magnification (Figure 7A). The numbers of vessels positive for CD68 and CD 206 on day 6 were higher than those on day 14 (Table 1). Wounds treated with DNH1 exhibited lower vessel numbers than the gauze group on days 6 and 14, whereas there was no difference between CD68 and CD206 on days 6 and 14. This tendency is similar to that previously reported [30,31]. Combined with the results of the wound closure rate shown in Figure 6C, it can be concluded that the number of CD68- and CD86-positive vessels in wounds treated with DNH1 was lower than those in wounds treated with gauze. Based on this observation, we concluded that DNH1 accelerated macrophage polarization from M1 to M2 before day 6, as illustrated in Figure 7C. Given that M2 macrophages promote the formation of mature blood vessels and secrete anti-inflammatory cytokines, their early polarization may contribute to improved healing outcomes. In summary, DNH1 not only shortened the wound healing time but also enhanced the quality of tissue regeneration.

## 3. Conclusions

In this study, we developed a DNH combined with hydrazone and Diels–Alder crosslinking to address the rapid degradation and weak mechanical properties of HA hydrogels. DNH was systematically tested and compared to HYH and DAH. HYH provided rapid crosslinking, whereas DAH contributed to the mechanical properties and extended the degradation time of the hydrogel. Among them, DNH1 exhibited optimal swelling properties, facilitating the efficient absorption of wound exudates while maintaining hydrogel integrity after swelling. To evaluate the therapeutic potential of DNH1, it was applied to a full-thickness rat skin wound model. The results demonstrated that DNH1 not only accelerated wound healing but also improved tissue regeneration. These findings highlight DNH as a promising candidate for wound dressings and offer an effective solution for enhanced wound repair.

## 4. Materials and Methods

### 4.1. Materials and Instrument

HA (20–50 kDa, HA0-01025) was purchased from Kishida Chemical (Osaka, Japan). Sodium periodate (NaIO_4_, 199-02401), ethylene glycol (054-00983), 2-morpholinethanesulfonic acid (MES, 343-01626), deuterium oxide (D_2_O, 99.8%, 049-34242), PBS, and isoflurane (099-06571) were purchased from Fujifilm Wako Chemicals Japan Corporation (Fukuoka, Japan). 4-(4,6-Dimethoxy-1,3,5-triazin-2-yl)-4-methylmorpholinium (DMTMM, D2919) and adipic dihydrazide (ADH, A0170) were purchased from TCI Tokyo Chemical Industry (Tokyo, Japan). Furfurylamine (F20009), hydroxylamine hydrochloride (255580), and fetal bovine serum (FBS, 173012) were purchased from Sigma-Aldrich (Tokyo, Japan). Maleimide-PEG-maleimide (Mal-PEG-Mal, HO022022-2K) was purchased from Funakoshi (Tokyo, Japan). NIH/3T3 clone 5611 (JCRB0615) was purchased from JCRB cell bank (Osaka, Japan). Dulbecco’s modified eagle’s medium (DMEM, SH30002.03) was purchased from Pall Life Sciences (Tokyo, Japan). Cell counting kit-8 (AJ006) was purchased from Dojindo Laboratories (Kumamoto, Japan). IHC-CD 31 (ab28364), CD-68 (ab125212), and CD 206 (ab234000) antibodies were purchased from Abcam (Cambridge, UK).

Water purification system (Direct-Q 3UV, Merck, Darmstadt, Germany); freezer dryer (WYELA FDU-1200, Tokyo Rikakikai, Tokyo, Japan); benchtop scanning electron microscope (JCM-7000, JEOL Ltd., Tokyo, Japan); tensile and compression test machine (LTTU-50 NB, Minebeamitsumi, Tokyo, Japan); ATR-FTIR spectrometer (4700, Jasco, Tokyo, Japan); ^1^H-NMR (JNM-ECZ400, JEOL Ltd., Tokyo, Japan); 8 mm skin biopsy puncture (Kai Industries, Tokyo, Japan); small animal anesthetizer (TK-36, Bio machinery, Seki-shi, Japan); inverted light microscope (CKX53, Olympus, Tokyo, Japan).

### 4.2. Synthesis of DNH

**Scheme 1:**
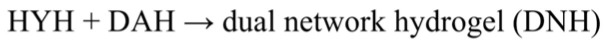
Synthesis of DNH.

HYH and DAH were mixed in a one-pot method (Scheme 1). OHA, ADH, HA-Furan, and Mal-PEG-Mal were treated with PBS as the solvent. The optimal conditions were obtained based on the synthesis of HYH and DAH.

**Scheme 2:**
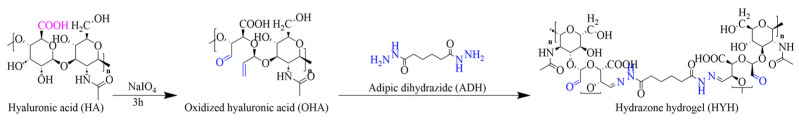
Synthesis of HYH.

HYH was prepared by mixing OHA and ADH (Scheme 2).

OHA was obtained using HA as a reactant by adding NaIO_4_ in the dark, following a previous report [32]. Briefly, HA (2.0 g) in 100 mL distilled water was kept at 4 °C overnight. NaIO_4_ (10.0 mL) in water (80 mg/mL) was added to HA solution and mixed under 25 °C for 3 h. A volume of 1396.4 μL of ethylene glycol was added to stop the reaction for 1 h under 25 °C. The solution was dialyzed with a dialysis bag (cutoff 1 kDa) against distilled water for 3 days to remove NaIO_4_ and ethylene glycol, during which the distilled water was changed three times each day. The dialysate was distributed into 6-well plates and pre-frozen at −80 °C overnight. OHA was obtained after lyophilization.

HYH was obtained by mixing OHA (192.0 μL) and ADH (48.0 µL) at 25 °C [33]. A total of 100 mg OHA in 1 mL PBS (pH 7.40) was used as a reactant. ADH in PBS at different concentrations (2, 4, 6, 8, 10, 20, 40, and 60 mg/mL) was evaluated. HYHs were named HYH1, HYH2, HYH3, HYH4, HYH5, HYH6, HYH7, and HYH8 following the related ADH concentrations.

**Scheme 3:**
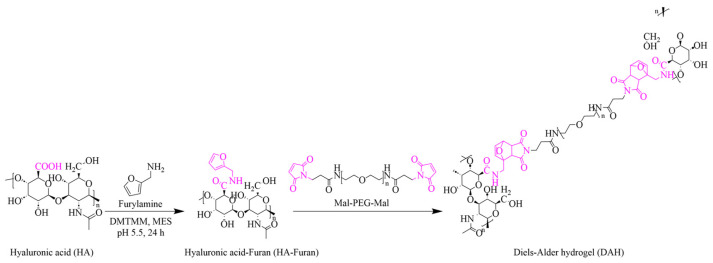
Synthesis of DAH.

DAH was synthesized by mixing HA-Furan with Mal-PEG-Mal in Scheme 3.

HA-Furan was prepared according to a previously reported method [34]. HA (2.0 g) dissolved in 100 mL MES buffer (100 mM, pH 5.50) was kept at 4 °C overnight. A total of 2.8 g DMTMM was added, followed by a dropwise addition of 486.0 μL furfurylamine after 10 min. The mixture was stirred for 24 h at 25 °C. The product was dialyzed against water for three days (cutoff: 1 kDa). HA-Furan was obtained by freeze-drying.

DAH was prepared by mixing HA-Furan and Mal-PEG-Mal in different volume ratios. Newly prepared lyophilized HA-Furan (100 mg/mL) and Mal-PEG-Mal (100 mg/mL) in PBS (pH 7.40) were mixed and kept overnight at 4 °C. DAH was formed at 37 °C overnight by vertexing [35]. Different volume ratios of HA-Furan and Mal-PEG-Mal (0.3, 0.5, 0.7, 1, 1.4, 2, 3, 5, 7, 9, and 11) were investigated and the results are listed in Table S3. Newly formed DAHs was named as DAH1, DAH2, DAH3, DAH4, DAH5, DAH6, DAH7, DAH8, DAH9, DAH10, and DAH11 following the different ratios between HA-Furan and Mal-PEG-Mal.

The DNH was obtained by mixing HA-Furan (100 mg/mL) and Mal-PEG-Mal (100 mg/mL), OHA (100 mg/mL, 64.0 μL), and ADH (8 mg/mL, 16.0 μL) with PBS as solvent. The sequence was added according to a previous report at 37 °C [17]. Different volume ratios of OHA and HA-Furan were evaluated (Table S1). The newly formed DNHs were named DNH1, DNH2, DNH3, DNH4, and DNH5.

### 4.3. Characterization of Hydrogel

#### 4.3.1. Determination of Oxidation Degree (OD) of OHA

The OD of OAH was determined by titration and is listed in Table S4 to determine the optimal ratio of HA to NaIO_4_. OHA (≈50 mg) was dissolved in 25 mL hydroxylamine hydrochloride solution (0.25 mmol/L, in H_2_O) and incubated for 5 h. The samples were titrated with NaOH (0.2 mol/L) until the pH increased to 9.97.

The reaction conducted during titration is shown below.

$$\begin{array}{c} \mathrm{HA-}(\mathrm{CHO})n+n\;H_{2}\mathrm{NOH}\cdot \mathrm{HCl}\to \mathrm{HA-}(\mathrm{CH=NOH})n+n\;\mathrm{HCl}\\ \mathrm{HCl}+\mathrm{NaOH}\to \mathrm{NaCl}+H_{2}O \end{array}$$

OD was calculated based on Equation (1):

OD (%) = Mw × C_NaOH_ × (V_OHA_ − V_blank_)/(2 × m_OHA_)× 100% (1)

where Mw = 400 g/mol, the monomeric molecular weight of OHA; C_NaOH_ = 0.2 mol/L; V_OHA_ is the NaOH volume (mL) consumed with OAH; V_blank_ is the NaOH volume (mL) consumed without OAH; and m_OHA_ is the mass of OHA in mg.

#### 4.3.2. Determination of OHA and HA-Furan Using FT-IR and ^1^H-NMR

ATR-FTIR was used to determine the aldehyde content of OHA, furan groups of HA-Furan, and the crosslinking bonds of HYH and DAH. The analyses were obtained from 400 to 4000 cm^−1^ with a resolution of 4 cm^−1^. Eight scans were performed for each sample.

^1^H-NMR was used to determine the OHA formation, the furan group of HA-Furan, and the modification degree of HA-Furan. The 0.6 mL of HA, OHA, and HA-Furan (10 mg/mL) were detected using D_2_O as the solvent. Chemical shifts were recorded.

#### 4.3.3. Characterization of Mechanical Properties and Gelation Time

The mechanical properties of the hydrogels were tested using a tensile- and compression-testing machine at a load of 50 N. The sample size was Ø = 7 mm and H = 5 mm. Stress–strain curve was plotted at a compression speed of 2 mm/s with the compression strain increasing from 0 to 70%. The maximum value indicates the maximum compressive stress. Young’s modulus was calculated based on the linear part of the stress–strain curve between 10% and 20%. The HYH results are presented in Table S2.

The gelation time was visually recorded. The time until no fluid remained in the inverted tube was recorded. The HYH results are presented in Table S2.

#### 4.3.4. Swelling Properties of Hydrogels Under Different pH

The samples for the swelling ratio of HYH, DAH, and DNH were prepared in a silicon mold (Ø = 8 mm, H= 0.5 mm), and the results were tested at varying pH. Samples were accurately weighted (*Wi*). The samples were immersed in PBS buffer with different pH values (3.0, 5.0, 7.0, 9.0, and 11.0, adjusted using HCl (1 M) and NaOH (1 M)), and the PBS was changed weekly. The treated samples were weighed (*Wt*) after the buffer solution was removed and wiped with paper towels. The swelling ratio *(SW)* was calculated using Equation (2):

*SW* = *Wt*/*Wi* (2)

#### 4.3.5. Characterization of Resilience

The resilience of HYH4, DAH3, and DNH1 cells was evaluated using tensile and compression testing. The resilience was recorded by measuring the height change in the samples after six compression cycles, with each cycle involving a compression strain of 60% and a compression speed of 2 mm/s.

#### 4.3.6. Characterization of Morphology of Hydrogels

The pore size and morphology of the hydrogels were determined using SEM. The hydrogels were placed in liquid nitrogen for 5 min and vertically removed. The samples were then freeze-dried and coated with gold for further analysis. Images of each sample were captured at ×500.

### 4.4. Cytocompatibility Testing of DNH1 In Vitro

The cytocompatibility of the DNH1 was evaluated using a leachate-based assay with mouse embryonic fibroblast NIH/3T3 cells. Hydrogel components were sterilized using 0.22 μm filters before hydrogel preparation. Hydrogels (Φ =16 mm, h = 0.5 mm) were soaked in a DMEM with 10% FBS to prepare leachates at two concentrations: 10% (100 µL hydrogel in 900 µL medium) and 20% (200 µL hydrogel in 800 µL medium). Prior to extraction, the medium was refreshed three times at 3 h intervals. The final leachates were collected after 24 h of incubation at 37 °C in 5% carbon dioxide. A control group with no hydrogel present (DMEM with 10% FBS only) was used as the control and designated as DNH-0%. NIH/3T3 cells were seeded at 6400 cells per well in 96-well plates and incubated with the leachates for 24 h. After removing the leachates, Cell Counting Kit-8 working solution (10% *v*/*v* in culture medium) was added and incubated for 2 h. Absorbance at 450 nanometers was measured using a microplate reader. Untreated cells served as the control. Cell viability was calculated according to Equation (3):

(3) $$Cell\;viability\;(\%)=\frac{As-Ab}{Ac-Ab}\times 100\%$$

where *As*, *Ab*, and *Ac* refer to sample, blank, and control absorbance, respectively. Cell morphology was observed using an inverted microscope.

### 4.5. Wound Healing Using DNH1

Eight male rats (6-week-old, Slc: S.D, SLC, Shizuoka, Japan) were acclimated to the laboratory conditions for 3 days. The average weight of all rats was 260 ± 10 g. All animals received humane care in accordance with the guidelines of the Animal Investigation Ethics Committee of Kyushu University. Animals (*n* = 2) were randomly divided into two groups according to treatment: gauze as the control group and DNH1 as the experimental group. Rats were housed in pairs (two rats per cage) in a temperature-controlled environment (25 ± 1 °C) with a 12 h light/dark cycle. The rats were provided with clean tap water and standard laboratory chow ad libitum. Anesthesia was induced using inhalational isoflurane during all surgical procedures to minimize pain and distress.

On day 0, the back of each rat was shaved. Double-circular full-thickness skin wounds were made on the back of each rat using a disposable 8 mm skin biopsy puncture. The wound circle was 9.5 cm from the tail and 1.5 cm from the mid-axis of the back of each rat. The skin was disinfected with a 50% (*v*/*v*) iodine solution in alcohol. Gauze or DNH1 was placed directly on the wound. The wound was additionally covered with a 5 cm × 10 cm piece of 3M Tegaderm to keep the dressing in place. The rat’s body was wrapped in an elastic bandage. The dressing was changed daily for the first 4 days. The dressing was changed every 2 days. The wounds were cleaned with a sterile 0.9% sodium chloride solution before each vehicle was changed. The DNH1 sheet was set to a diameter of 20 mm and 1.5 mm in thickness for the first 6 days. The DNH1 sheets were reduced to a diameter of 10 mm before sacrifice.

The wound closure rate was determined using wound photo analysis. The wound area of each rat was analyzed using ImageJ software (version 1.54d). The wound closure rate was expressed as a percentage of the original area on day 0 using Equation (4) [36]:

Wound closure rate (%) = [1 − wound area (day t)/wound area (day 0)] × 100%(4)

where t represents the time point of wound healing.

The rats were sacrificed on days 6 and 14 after wound healing. Wound sites on the healthy skin were excised and fixed in 10% formalin. Tissues were treated with a graded alcohol series and xylene and then embedded in paraffin. Central wound sections (4 mm) were mounted on glass slides and stained with H&E or with IHC for CD 31, CD-68, and CD 206. Images of each sample were acquired using an optical microscope with the ×20, ×100, and ×200 objectives.

### 4.6. Statistical Analysis

All experimental data were expressed as mean ± standard division (S.D.). Statistical significance was determined using one-way ANOVA, followed by Tukey’s Honest significant difference (HSD) test for multiple comparisons.

## Figures and Tables

**Table 1:** Quantification of histological wound samples obtained on days 6 and 14 (*n* = 2, the value indicates mean ± S.D.).

	Day 6		Day 14	
	Gauze	DNH1	Gauze	DNH1
Epidermis thickness (μm)	77.33 ± 4.33	64.59 ± 32.12	47.98 ± 3.27	45.6 ± 3.83
Dermis thickness (μm)	1198.99 ± 636.69	1126.83 ± 23.31	993.70 ± 90.29	1323.16 ± 654.28
Vessel number (-)	116.5 ± 37.48	36.00 ± 21.21	75.50 ± 3.54	38.50 ± 2.12
CD68 (-)	172 ± 8.49	106.5 ± 71.42	28.5 ± 10.61	24.50 ± 26.16
CD206 (-)	234 ± 50.91	135 ± 94.75	19.50 ± 3.54	25.5 ± 13.44

## Data Availability

Data are contained within the article and Supplementary Materials.

## Supplementary Materials

The following supporting information can be downloaded at: https://www.mdpi.com/article/10.3390/gels11040266/s1, Table S1: Volume ratios of HA-Furan, Mal-PEG-Mal, OHA, and ADH during DNH preparation; Table S2: HYH properties (*n* = 3, the value indicates mean ± S.D.; Table S3: Volume ratios of HA-Furan and Mal-PEG-Mal during DAH preparation; Table S4: Effect of the molar ratio between HA and NaIO_4_ on gelation time (*n* = 3, the value indicates mean ± S.D.).

## Abbreviations

The following abbreviations are used in this manuscript:

DNH	Double network hydrogel
HA	Hyaluronic acid
ECM	Extracellular matrix
HYH	Hydrazone hydrogel
OHA	Oxidized hyaluronic acid
ADH	Adipic dihydrazide
DAH	Diels–Alder hydrogel
HA-Furan	Hyaluronic acid with furan group
Mal-PEG-Mal	Maleimide-PEG-maleimide
