# Supplementary material for: Fast Wound Healing with a New Functional Hyaluronic Acid Dual Network Hydrogel
Source: Gels. 2025 Apr 3;11(4):266. doi: 10.3390/gels11040266 (PMC12027019; doi:10.3390/gels11040266)
Supplement: Supplementary file 1 [file gels-11-00266-s001.zip › gels-3539452-supplementary.pdf]

Table S1. Volume ratios of HA-Furan, Mal-PEG-Mal, OHA, and ADH during DNH preparation.

Sample name	HA-Furan volume (μL)	Mal-PEG-Mal volume (μL)	OHA Volume (μL)	ADH Volume (μL)
DNH1	57.6	82.4		
DNH2	70.0	70.0		
DNH3	93.3	66.7	64.0	16.0
DNH4	106.7	53.3		
DNH5	120.0	40.0		

Table S2. HYH properties (n=3, the value indicates mean ± S.D.)

Sample name	Maximum strain	Maximum stress (kPa)	Young's modulus (kPa)	Gelation time (s)
HYH1	No data	No data	0.88±1.62	10.00±1.00
HYH2	74%	154.46	12.85±4.08	8.67±1.53
HYH3	88%	712.24	18.98±1.90	8.67±1.53
HYH4	87%	654.25	28.87±1.92	10.00±1.73
HYH5	87%	445.96	18.89±2.67	13.00±1.00
HYH6	74%	152.09	17.66±4.36	21.00±1.00
HYH7	75%	99.05	9.99±2.45	47.67±7.23
HYH8	No data	No data	6.07±1.63	148.00±10.58

Table S3. Volume ratios of HA-Furan and Mal-PEG-Mal during DAH preparation

Sample name	Volume ratio of HA-Furan: Mal-PEG-Mal	HA-Furan volume (μL)	Mal-PEG-Mal volume (μL)
DAH1	0.3	60.0	180.0
DAH2	0.5	80.0	160.0
DAH3	0.7	100.0	140.0
DAH4	1	120.0	120.0
DAH5	1.4	140.0	100.0
DAH6	2	160.0	80.0
DAH7	3	180.0	60.0
DAH8	5	200.0	40.0
DAH9	7	210.0	30.0
DAH10	9	216.0	24.0
DAH11	11	220.0	20.0

Table S4. Effect of the molar ratio between HA and $NaIO_4$ on gelation time (n=3, the value indicates mean ± S.D.)

Molar ratio of HA: $NaIO_4$	OD	Gelation time (s) [a]
2:1	14%	24.67±0.58
4:3	28%	21.00±1.00
1:1	43%	11.67±0.58

[a] Gelation time was recorded during the synthesis of HYH with OHA and ADH (20 mg/mL) as reactants, and the 4:3 ratio was selected because of its suitable gelation time. Increasing the rate resulted in shorter gelation time, making further operations difficult.
